# Emergency balloon dilation or stenting of critical coarctation of aorta in newborns and infants: An effective interim palliation

**DOI:** 10.4103/0974-2069.58311

**Published:** 2009

**Authors:** Edwin Francis, S Gayathri, Balu Vaidyanathan, B R J Kannan, R Krishna Kumar

**Affiliations:** Department of Pediatric Cardiology, Amrita Institute of Medical Sciences and Research Center, Kochi, India; 1 Vadamalayan Hospitals, Chokkikulam, Madurai, India

**Keywords:** Aortic coarctation, catheter intervention, left ventricular dysfunction

## Abstract

**Background:**

Management of native uncomplicated coarctation in neonates remains controversial with current evidence favoring surgery. The logistics of organizing surgical repair at short notice in sick infants with critical coarctation can be challenging.

**Methods and Results:**

We reviewed data of 10 infants (mean age of 2.9 ±1.6 weeks) who underwent catheter intervention for severe coarctation and left ventricular (LV) dysfunction between July 2003 and August 2007. Additional cardiac lesions were present in 7. Mean systolic gradient declined from 51±12 mm Hg to 8.7±6.7 mm Hg after dilation. The coarctation segment was stented in five patients. Procedural success was achieved in all patients with no mortality. Complications included brief cardiopulmonary arrest (n =1), sepsis (n = 1) and temporary pulse loss (n = 2). LV dysfunction improved in all patients. Average ICU stay was 5±3.4 days and hospital stay was 6.5±3.4 days. On follow-up (14.1±10.5 months), all developed restenosis after median period of 12 weeks (range four to 28 weeks). Three (two with stents) underwent elective coarctation repair, two underwent ventricular septal defect (VSD) closure and coarctation repair and one underwent pulmonary artery (PA) banding. Two patients who developed restenosis on follow-up were advised surgery, but did not report. Two (one with stent) underwent redilatation and are being followed with no significant residual gradients.

**Conclusion:**

Balloon dilation ± stenting is an effective interim palliation for infants and newborns with critical coarctation and LV dysfunction. Restenosis is inevitable and requires to be addressed.

## INTRODUCTION

It is not uncommon for newborns and young infants with severe coarctation of aorta to present with cardiogenic shock, metabolic acidosis and end organ dysfunction when the ductus arteriosus closes.[1] Response to prostaglandin can be unpredictable particularly among newborns who present beyond two weeks of age. In these critically ill infants, clinical condition is likely to worsen unless the obstruction is relieved immediately.[1] Many associated defects are common and include VSD, bicommisural aortic valve and Shone’s syndrome.[2] It is difficult to organize emergency surgery, particularly in centers in the developing world. Critically ill infants with end organ injury pose additional challenges. The role of catheter intervention for interim palliation of this difficult subset has not been clearly defined.[3] There is very little published information on the role of stenting in these patients. Because of concern regarding future repair, stents are traditionally avoided. However, some infants respond poorly to balloon dilatation alone, particularly when isthmus is hypoplastic.[3] We present our experience with emergency balloon dilation and stenting in newborns and infants with severe coarctation associated with LV dysfunction. Immediate and short term follow-up results are discussed.

## METHODS

After obtaining permission from the medical records department we retrospectively analyzed the data of all infants with severe coarctation of aorta and LV dysfunction who presented to us with either cardiogenic shock or heart failure with respiratory distress severe enough to merit admission to the intensive care unit from January 2003 to December 2007. We identified 10 patients who underwent emergency balloon dilatation with or without stenting. Patients who were hemodynamically stable with normal left ventricular function were excluded. Clinical and hemodynamic profile, associated cardiac defects, procedural details and complications, immediate and short term follow-up details were recorded.

### Details of procedure

**Figure 1:**
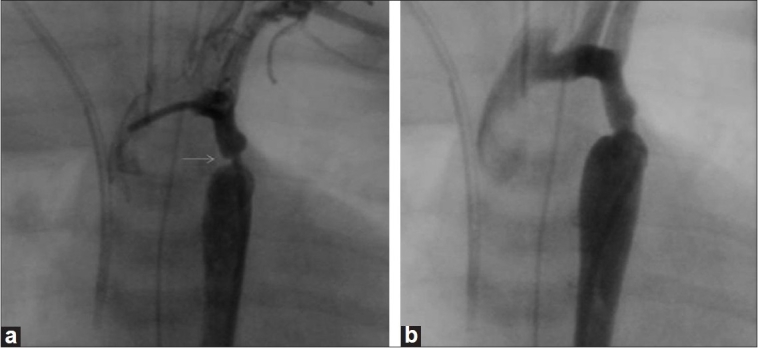
(a) Pre and (b) post angiographic frames of a newborn coarctation stenting. Arrow indicates the site of narrowing

**Figure 2:**
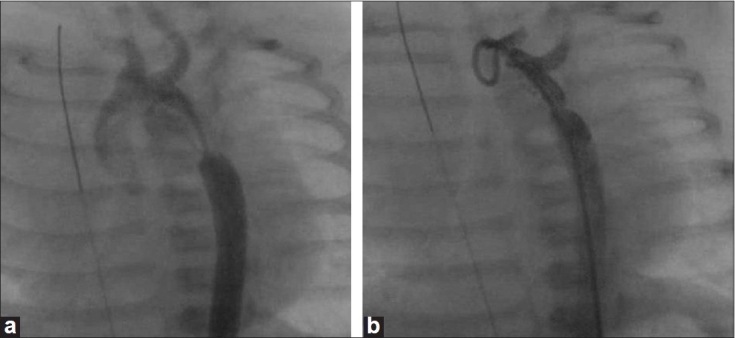
(a) Angiographic frames showing discrete coarctation and (b) final angiogram showing well open coarctation segment

After explaining the details of the procedure, complications and alternative options, an informed consent was taken from parents. Conscious sedation was given with ketamine infusion (50μgm/kg/min) in all except three cases in whom prior mechanical ventilation was instituted. A 4F pediatric introducer sheath was inserted in one of the femoral arteries and 100 U/kg of heparin administered. A 4F right coronary catheter with 0.018 Terumo wire was used to cross the coarctation segment. No difficulty was encountered during attempts to cross the segment. Pressure gradients were recorded across the coarctation segment. An aortic angiogram straddling coarctation segment was done using a 4 F pigtail in both posterior-anterior and lateral views [Figure 1a and 2a]. Isthmus and transverse arch measurements were made. The balloon diameter for dilatation was determined by the echocardiographic or angiographic (whichever was larger) measurement of the isthmus. The narrowest coarctation diameter was not used to determine balloon size. Balloon was upsized if residual gradients were present, but did not exceed distal transverse arch dimension. Coronary and renal stent balloons were used for dilatation using an indeflator with a pressure gauge. The balloon dilatation was graded and the nominal inflation pressures recommended for the balloons were not exceeded. Pressure gradients were recorded across the dilated/stented segment and repeat angiograms [Figure 1b and 2b] were done to assess the efficacy of the procedure.

The decision to stent the coarctation segment depended on the aortic arch anatomy, degree of arch hypoplasia and associated cardiac lesions. The decision was made after angiography itself. The criteria for stenting were not predefined and not uniform. In general stent was used when the isthmus was hypoplastic (Z scores < -2; Figure 2a). A visual assessment of the relative size of the transverse arch and the isthmus in comparison with the ascending aorta was also a factor in determining the presence of hypoplasia.[2] The presence of associated conditions which made immediate surgical intervention more difficult, such as Shones complex, lowered our threshold for stenting to enable a lasting palliation. Stent was deployed primarily and not after balloon dilatation. Balloon dilatation alone was preferred when the coarctation was thought to be discrete with a good sized isthmus (Z scores> -2).

We did not prefer any specific manufacturer or design for the stent. Essentially any premounted bare metal renal or coronary stent of appropriate diameter (4-5 mm) with a length of 8-13 mm was used. These stents are commonly stocked in cardiac catheterization laboratories with a large adult case volume. The following stents were most commonly used: coronary Zeta stent (Abbott Vascular), Racer renal stent (Medtronic AVE), prolink or prostar stents (Vascular Concepts, Bangalore, India). All stents were deployed at the recommended pressure. If clear landmarks were seen on angiography, stents were deployed without the use of a long sheath. Otherwise, the short sheath in the groin was replaced by a 4F long sheath (Cook Inc, IN, USA). This allowed frequent check angiograms to guide stent placement. After balloon dilation or stent deployment final angiograms and pullback gradients were obtained. Patients were discharged as early as the clinical condition dictated and were followed up in the outpatient clinic every two weeks. No anticoagulation or antiplatelet agents were used.

## RESULTS

During the study period 51 infants underwent surgical repair of coarctation at our institute. Balloon dilatation or stenting was performed in 10 patients who presented in a critical state with respiratory distress and/or hemodynamic compromise. Of the 10 patients studied, nine were neonates. Their mean age was 2.9±1.6 weeks mean weight was 2.9±0.7 kg. While seven patients had discrete post subclavian coarctation; three had associated arch hypoplasia. Associated conditions included VSD in three patients, bicommissural aortic valve with aortic stenosis in one patient, Shone’s complex in one and hypoplastic mitral annulus (Z score < - 2) in one. None of the patients had features of Turner’s syndrome. There was significant hemodynamic compromise in eight patients. Hemodynamic compromise was defined as significant systemic hypotension resulting in tissue hypoperfusion; six required inotropes, one patient improved with fluids, another with prostaglandin and other general supportive measures. At admission, additional problems included metabolic acidosis in four patients, renal failure in one and hepatic dysfunction in one. Mechanical ventilation was required for stabilization in three patients. All patients successfully underwent cardiac catheterization with dilatation of the coarctation segment with no procedural mortality. Based on arch anatomy and associated cardiac lesions, five patients underwent stenting with coronary/renal stents. All three patients with tubular hypoplasia of arch underwent stenting. Fluoroscopy time was 12 ± 3 minutes for the entire group of 10 infants. The gradients across coarctation reduced from 51±12 mm Hg to 13±4 mm Hg. Complications included transient cardio-respiratory arrest in one patient during the procedure, gram negative sepsis in one and temporary pulse loss in lower limb requiring heparin infusion in two. Left ventricular systolic function normalized (LVEF> 50%) in all patients within 24 – 48 hours. The mean ICU stay was 5±3.43 days and the mean total hospital stay was 6.5±3.4 days. On follow-up, reintervention was required after a median interval of 12 weeks (four to 28 weeks). Reintervention was advised when clinical or echocardiographic gradients across the coarctation segment exceeded 20 mm Hg. Three patients (two with stents) underwent elective coarctation repair. Two patients underwent VSD closure and coarctation repair and another one who had a large unrestrictive posterior muscular VSD underwent PA banding. Two patients on four months follow-up showed significant restenosis and are awaiting surgical repair. All patients were offered surgery. Redilatation was recommended for those who could not be offered surgery because of logistic or economic reasons. Two patients underwent redilatation for restenosis. One of these had received a five mm stent that was re dilated with a six mm balloon after 16 weeks; the other patient (initially underwent dilation with a four mm balloon) underwent redilatation 17 months later with a 10 mm balloon. Both are being followed up (32, 28 weeks) with no significant residual stenosis (< 10 mm Hg gradient across the coarctation segment). Patient characteristics, procedural details and follow up are summarized in Table 1.

**Table 1:** Baseline characteristics, procedural and follow-up details of 10 infants undergoing balloon dilation or stenting as interim palliation for coarctation

Age (wks)	Wt (kg)	Isthmus (mm)	Transverse arch (mm)	Associated lesions	Intervention	Residual gradient (mmHg)	Complications	ICU stay (days)	Hospital stay (days)	Follow up (months)	Outcome
4	3	4	4.5	Shone’s syndrome	5 × 13 mm stent	8	Transient pulse loss	5	7	32	Stent redilation done after 3 months
1	2.8	3	3.2	Hypo plastic LV, hypo plastic transverse arch	4 × 10 mm stent	15	-	7	12	4	Refused surgery, no further follow up
3	3.5	4.3	4.8	Bicuspid aortic valve with aortic stenosis	Balloon (4 × 15 mm)	Nil	-	1	4	28	Redilation done twice
3	2.7	4.3	6.3	Nil	Balloon (6 × 20 mm)	Nil	-	1	6	6	Surgery planned
6	3.2	3.2	3.5	VSD, hypo plastic arch	4 × 9 mm stent	Nil	-	2	4	2	PA banding done then lost to follow up
4	0.97	2.7	3.8	VSD	Balloon (5 × 20 mm)	8	Transient cardiac arrest during procedure	2	5	9	VSD closure and CoA repair done 3 months later
1	3.1	3	7.5	VSD	4 × 8 mm stent	16	Sepsis	5	13	12	VSD closure and CoA repair 3 months later
4	3.1	3.4	3.6	Hypo plastic arch	4 × 10 mm stent	15	-	3	5	24	CoA repair 3 months later
1	3	5	6	Nil	Balloon (6 × 15 mm)	15	-	3	6	8	CoA repair one month later
2	3.3	3.7	4.8	Nil	Balloon (5 × 15 mm)	10	-	2	3	16	CoA repair 2 months later

CoA - Coarctation; LV - Left ventricle; PA - Pulmonary artery; VSD - Ventricular septal defect

## DISCUSSION

Balloon dilatation with or without stenting of native coarctation is an accepted mode of treatment in older children and adults with results comparable to that of surgery. However, balloon angioplasty for native coarctation of aorta in neonates and infants remains controversial. Problems related to balloon angioplasty in neonates and young infants include residual gradients when associated with long segment coarctation or associated arch hypoplasia, higher incidence of restenosis and aneurysm formation compared with surgical management. Surgery is considered treatment of choice for coarctation of aorta in neonates and young infants. In accordance with our institutional policy, balloon angioplasty with or without stent implantation was offered as an interim palliation for sick infants with coarctation and LV dysfunction or those with complex associated lesions, whenever emergency surgical repair was not possible. Immediate outcome was excellent in all with significant reduction in coarctation gradient. There was symptomatic improvement in all patients and three of them who required ventilator support because of hemodynamic instability and acidosis recovered rapidly. Postprocedure ventilatory requirement was 22±12 hours. Left ventricular function improved in all within 24 to 48 hours. This clinical improvement and relief of obstruction could be achieved with no mortality and minimal morbidity in this subset of sick patients.

Neonatal coarctation repair when associated with other cardiac lesions or left ventricular dysfunction continues to have finite mortality and high morbidity.[3‐5] Recently Fesseha *et al*. reported good results with surgical correction of neonates with aortic coarctation and cardiogenic shock.[6] This study did not include patients with complex forms of congenital heart disease. Organizing emergency surgery can be challenging in many centers, especially in the developing world because of limitations of human and material resources. In complex congenital cardiac anomalies surgical options of primary repair are limited. The role of transcatheter palliation is not well defined. In a recent publication stent angioplasty was used successfully in four infants with critical native coarctation who had associated co-morbidities which contraindicated surgery.[7] Since the early report of balloon angioplasty by Labibidi in 1984, many reports of balloon angioplasty or stenting in the newborns or young infants with varying results have been published with few operators recommending the procedure, while others preferring surgery against angioplasty.[8‐12] In our series, all patients had significant reduction in gradients with only a few procedural complications. Diameter of balloon for dilatation was selected depending on the isthmus dimension and maximum balloon size was limited by the dimensions of transverse arch. The narrowest diameter of the coarctation segment was not considered in determining the balloon size. This is different from the previous recommendations by Rao *et al*. wherein the maximum balloon diameter was determined by the descending aortic dimension.[9,10] Good immediate relief of gradients can be achieved with balloon dilatation alone in majority of cases, but early restenosis is well known[12] Therefore some of the patients (those with associated cardiac lesions which made them high risk candidates for immediate surgical repair and some of the infants with hypoplastic arch) underwent stenting of the coarctation segment. The shortest stent length was used to minimize interference during later surgical repair and to decrease the prospect of jailing the left subclavian artery. Redington *et al*. reported restenosis in majority of patients after balloon angioplasty in 10 neonates.[13] The results did not support the use of balloon angioplasty in neonates with coarctation. Similar results were reported by Park *et al*.[14] More recently Patel *et al*. reported 17 hemodynamically stable patients with median age of 3 months who had undergone angioplasty for coarctation.[15] Restenosis rate was 41% at 2.7 years of follow up. Unlike the previous reports patients in our study were either hemodynamically unstable and/or associated with complex cardiac anomalies in which surgical approach carried significant morbidity and mortality. Stents were used in five of our patients, mostly in those with hypoplastic arch.

There was no aneurysm formation detected in the short term follow-up with echocardiography alone. As expected, all patients developed re-coarctation on follow up. Reintervention was required after a mean period of four months after the initial procedure. The major limitation of conventional stents in small infants is that they prevent further vessel growth, require redilatation, and later, surgical removal. Biodegradable magnesium stents or growth stents may overcome these limitations and can dramatically change the treatment of these patients.[16] As of now there are only limited reports of these stents being used for infants and newborns[17]

### Study limitations

This is a retrospective study with a relatively small number of patients with considerable diversity in disease and associated lesions. The decision to do balloon dilation vs. stent was somewhat subjective and not based on uniform predefined criteria. Late follow-up computerized tomography scans or magnetic resonance imaging evaluations will be required to assess the repaired segment to rule out aneurysm formation.

## CONCLUSION

This report describes the feasibility of balloon dilatation with or without stenting as an effective interim palliation for sick infants and newborns with severe coarctation and LV dysfunction. Restenosis is inevitable and needs to be addressed during follow-up.
